# 

**Published:** 1889-06

**Authors:** 


RECEIPTED FOR 1889.—Drs. L M Wood, N B Null, J A Agnew, W C Kiser, J E Martin, E W Lane, L W Mobley, Jno. Hardeman, J T Pou, Wm. Grimes, Jno. S Moreton, A L Simon, R S Phillips, E J Stubbs, E L Haynes, T A Christian, T S Fox, to May; R H Coleman, to June.

